# N-glycosylation enables high lateral mobility of GPI-anchored proteins at a molecular crowding threshold

**DOI:** 10.1038/ncomms12870

**Published:** 2016-09-19

**Authors:** Andreas J. W. Hartel, Marius Glogger, Nicola G. Jones, Wasim Abuillan, Christopher Batram, Anne Hermann, Susanne F. Fenz, Motomu Tanaka, Markus Engstler

**Affiliations:** 1 Department of Cell and Developmental Biology, Theodor-Boveri-Institute, Biocenter, University of Würzburg, Würzburg 97074, Germany; 2 Physical Chemistry of Biosystems, Institute of Physical Chemistry, University of Heidelberg, Heidelberg 69120, Germany; 3 Institute for Integrated Cell-Material Science (WPI iCeMS), Kyoto University, Kyoto 606-8501, Japan

## Abstract

The protein density in biological membranes can be extraordinarily high, but the impact of molecular crowding on the diffusion of membrane proteins has not been studied systematically in a natural system. The diversity of the membrane proteome of most cells may preclude systematic studies. African trypanosomes, however, feature a uniform surface coat that is dominated by a single type of variant surface glycoprotein (VSG). Here we study the density-dependence of the diffusion of different glycosylphosphatidylinositol-anchored VSG-types on living cells and in artificial membranes. Our results suggest that a specific molecular crowding threshold (MCT) limits diffusion and hence affects protein function. Obstacles in the form of heterologous proteins compromise the diffusion coefficient and the MCT. The trypanosome VSG-coat operates very close to its MCT. Importantly, our experiments show that N-linked glycans act as molecular insulators that reduce retarding intermolecular interactions allowing membrane proteins to function correctly even when densely packed.

## Abstract (web summary)

How molecular crowding affects membrane protein diffusion and function is not known. Here the authors measure diffusion of variant surface glycoprotein on trypanosomes and discover a molecular crowding threshold that limits diffusion, and find that N-linked glycans help to prevent retarding intermolecular interactions.

## Graphical abstract

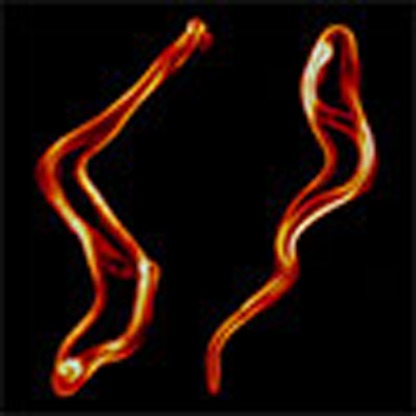

Biological membranes are vital components of all living systems, as they form the outer boundary of cells and enclose their internal compartments. They consist of a lipid bilayer that imparts a fluid character, and a multitude of membrane-associated proteins with a range of functions.

The translational diffusion of proteins in biological membranes has been described theoretically by Saffman and Delbrück within the framework of a continuum approach^1^. In their landmark model, the diffusion coefficient of a particle embedded in a membrane is largely determined by the viscosity and thickness of the bilayer and has only a weak dependence on the radius of the membrane-spanning domains. The translational diffusion coefficient of a cylindrical particle (radius *R*_p_<<*h μ*_m_*/μ*_w_) immersed in a quasi-two-dimensional (2D) continuum bounded by less viscous medium (*μ*_w_<<*μ*_m_) is


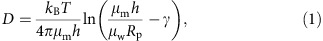


where *k*_B_ is the Boltzmann constant, *T* the temperature *μ*_w_ and *μ*_m_ are the bulk viscosities of the surrounding medium and the lipid membrane, respectively. The height of the particle *h* equals the thickness of the bilayer, and *γ* is the Euler's constant (*γ*=0.5772). The Saffman–Delbrück (SD) model was verified experimentally for protein and peptide diffusion in model membranes^2^,^3^,^4^,^5^,^6^,^7^. A more general, but mathematically more complex model, valid for arbitrary viscosities of the membrane and surrounding medium as well as a larger range of *R*_p_, was provided by Hughes *et al*.^8^ Today, a reliable analytical approximation of this model is available thus facilitating its use^9^.

To model the lateral diffusion of lipids and proteins in contact with asymmetric environments, such as a rigid substrate, Evans and Sackmann extended the continuum model by taking asymmetric boundary conditions and the resulting friction on the membrane into account^10^.

The above models are based on the assumption that sparsely dispersed proteins undergo free random walks and therefore do not account for the potentially high protein density on biological membranes^11^. Other theoretical studies have considered higher surface densities. They predict that the lateral diffusion of a given protein is hindered by the presence of additional proteins, which act as obstacles^12^,^13^,^14^. Experiments both in crowded model membranes^5^,^15^,^16^,^17^ and living cells^18^,^19^ reported reduced normal diffusion, emergence of sub-diffusion, obstructed diffusion or clustering as a function of protein density.

Coarse-grained molecular dynamics simulations in crowded membranes predicted sub-diffusion on the microsecond timescale and reduced diffusion at experimentally accessible scales^20^,^21^,^22^. Recently, a study based on dissipative particle dynamics suggested a relation between the friction coefficient introduced by crowding and the diffusion constant. In this model for dilute and semi-dilute conditions (occupied area fraction<0.4) the contribution of the total protein density to the friction coefficient is a polynom of second order^23^.

The SD model and the subsequent mathematical calculations only take into account the membrane-spanning domain of a protein. The protein part that protrudes above the membrane is deemed to have no effect on diffusion, since the viscosity of the surrounding medium is much lower than the viscosity of the lipid bilayer. However, we and others could show that changes in the extra-membrane domain of a protein significantly changes the resulting diffusion coefficient^24^,^25^,^26^,^27^. In this work, we address another parameter that cannot be captured by these simple models: the effect of molecular crowding in the semi-dilute approximation and beyond, which has so far not been studied in a natural system.

This may be due to the fact that the vast majority of cells exhibit a great diversity of membrane proteins, each of varying abundance and with different properties. The sizes of the various proteins differ, as do the mode and degree of membrane attachment. Thus, a natural cell system with reduced complexity, high flexibility, yet maximum conservation is required to assess the potential impact of molecular crowding on biological membranes in a comparative and quantitative manner. We suggest that the surface coat of African trypanosomes, the causative agents of sleeping sickness in humans and Nagana in cattle, provides such a model. The unicellular parasites are covered with a dense layer of variant surface glycoproteins (VSGs). About 95% of all surface proteins are VSGs^28^. An extracellular matrix does not exist^29^. There is strong selective pressure for the VSG coat to maintain a sufficiently high density, as it functions in shielding other membrane proteins from recognition by the host's immune system^30^. African trypanosomes possess several hundred different VSG genes, of which only one is expressed at any given time. Random switches to the expression of a new VSG allow escape from the mammalian immune response by antigenic variation^31^. Thus, there is selective pressure for a VSG repertoire that is not only diverse in sequence but also shows structural conservation. Although VGSs are thought to be structurally similar^32^,^33^, they are immunologically clearly distinct. In addition, VSGs are glycosylphosphatidylinositol (GPI)-anchored proteins^34^, which means they are attached only to the outer leaflet of the plasma membrane by the GPI moiety. As a consequence, the interaction between the proteinaceous part and the plasma membrane is minimized when compared with transmembrane domains. Since the core structure of the membrane anchor of all VSG types is identical^35^, true comparative studies to elucidate the role of the free protein part on diffusion become possible.

Here we present a systematic study on the influence of protein lateral density and N-glycosylation on the diffusion of the GPI-anchored VSG first on the trypanosome cell surface, and second in a more defined artificial supported membrane system. Our data indicate the presence of a molecular crowding threshold (MCT) for lateral diffusion. The MCT is not only determined by the lateral protein density but also by the biochemical properties of the proteins. We found that the trypanosome cell surface acts remarkably close to its MCT. This threshold is modulated by N-glycosylation.

## Results

### The diffusion behaviour of different VSGs is comparable

We established both an *in vivo* and an *in vitro* experimental system for direct comparison of the diffusion of VSGs in natural and artificial surface coats. First, we developed a straightforward method to immobilize the motile flagellate parasites^36^. Cells were incubated in a custom-made observation chamber in trypanosome dilution buffer (TDB) or PBS, containing ∼6% (w/v) type A gelatin. At 37 °C, this solution is fluid, allowing normal cellular movement. With decreasing temperature the gelatin becomes solid, resulting in complete immobilization of trypanosomes at 20 °C. It should be noted that this process is fully reversible and allows immobilization of various trypanosome strains and species for several hours without any negative impact on subsequent growth of the trypanosomes after their release from the solid matrix (Supplementary Note 1 and Supplementary Fig. 1).

Although it appears common knowledge that the diffusion of different VSGs (MW=50–60 kDa per monomer) is similar, no comparative measurements have been reported to date. The diffusion constant for one VSG has, however, been determined^37^. Therefore, the diffusion coefficients and mobile fractions of a variety of fluorescently labelled VSGs were determined on living cells by fluorescence recovery after photobleaching (FRAP) measurements. This experiment involved five trypanosome strains, expressing representative VSGs, which contain either one, two or three N-glycans^38^. The diffusion coefficients were found to lie within a narrow range, between 0.02 and 0.03 μm^2^ s^−1^. The mobile fractions were ∼80%, indicating that a very high fraction of VSGs moved freely on living trypanosomes (Table 1). This shows that despite marked differences in protein primary structure, domain architecture and number of N-glycans, the VGSs in fact display very similar diffusion parameters. Note, that the immobile fraction is only immobile on the timescales used for the experiment not, however, on longer timescales. In the next experiment, we compared the results obtained from living trypanosomes with purified VSGs inserted into artificial supported membranes. For protein isolation, we adopted a protocol that uses trifluoroacetic acid extraction and reversed-phase high-performance liquid chromatography^39^. Four types of purified mfVSGs were fluorescently labelled using succinimidyl ester chemistry and incorporated into preformed lipid bilayers, which were made by spreading small unilamellar lipid vesicles onto glass substrates. Equal amounts of different mfVSGs were allowed to incorporate into the lipid bilayers by incubation under standardized conditions. The diffusion coefficients determined for the different VSGs in the supported membranes were virtually identical (Table 1), which is in good agreement with the *in vivo* analysis. Furthermore, the diffusion coefficients of around 1.0 μm^2^ s^−1^ are comparable to those published for other GPI-anchored proteins in artificial lipid bilayers^40^, which emphasizes that VSGs are representative, model GPI-anchored proteins.

The diffusion coefficient of VSGs in supported membranes was significantly larger than on living trypanosomes. Several factors might be responsible, for example molecular crowding of VSG, the presence of heterologous proteins on the trypanosome cell surface, the differences in the lipid composition of the model and natural membrane, or high extracellular viscosity originating from gelatin embedding. The latter was ruled out by measuring the diffusion coefficients of VSG in supported membranes in the presence and absence of 6% (w/v) gelatin; no negative effect on the diffusion of VSG was observed (Supplementary Table 1). Therefore, we investigated the other feasible possibilities in separate sets of experiments.

### VSG coats operate close to a molecular crowding threshold

**Figure 1:**
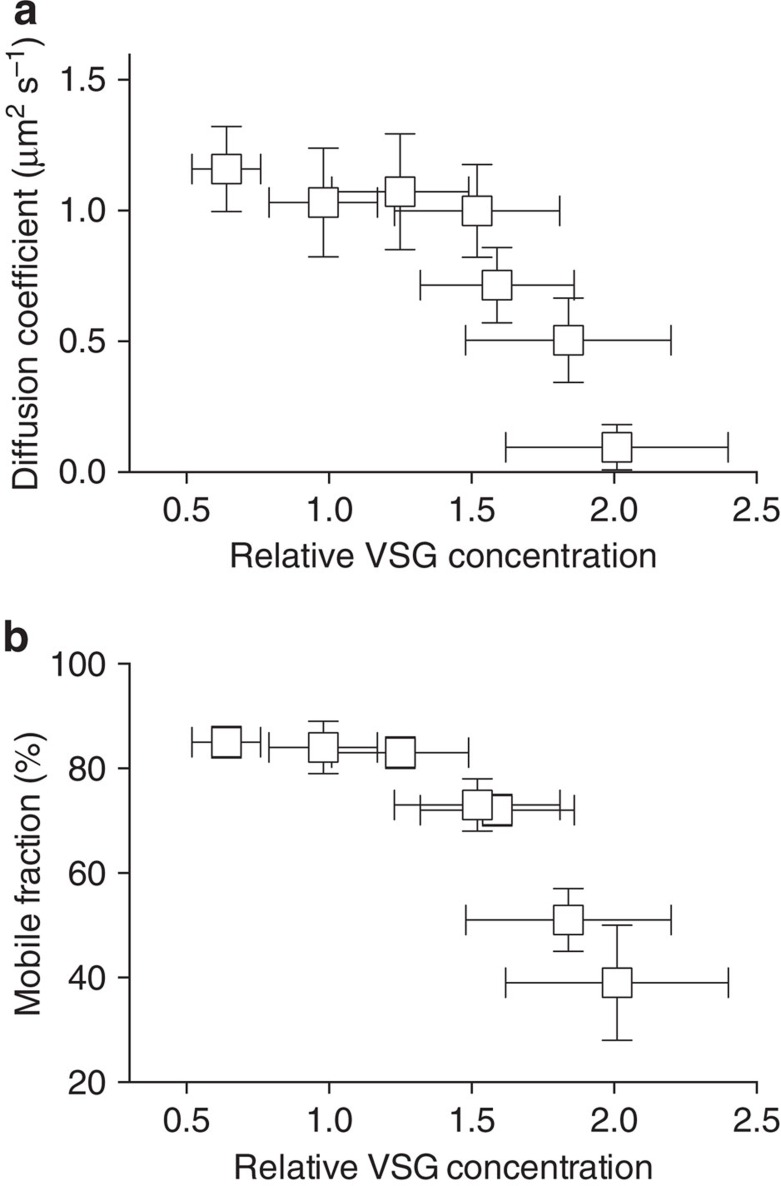
The VSG coat operates close to its MCT. The impact of lateral protein density on the diffusion of VSG was quantified in supported membranes. Diffusion coefficients (**a**) and mobile fractions (**b**) are displayed as a function of the VSG concentration in relation to the concentration on the trypanosome cell surface. A relative concentration of 1 corresponds to the concentration *in vivo.* The error bars in *y* (diffusion coefficient) represent the standard deviation of the mean, while the error bars in *x* (relative VSG concentration) depict the range.

We developed a system that allows precise adjustment of VSG density within supported membranes. The aim was to perform a direct comparison of the concentration of the proteins in artificial lipid bilayers with that on living trypanosomes. The exact number of cells in exponentially growing trypanosome cultures was determined by a linear dilution series and the corresponding amount of VSG was measured by immuno-detection and near infrared fluorescence scanning of dot blots. Next, we quantified the amount of VSG incorporated into solid supported lipid bilayers after fusion of proteoliposomes to lipid monolayers, which had been prepared using a Langmuir film balance (see ‘Methods' section for more details). Figure 1 shows the diffusion of VSGs at various lateral densities. Remarkably, the experiments revealed that a very high and controllable degree of molecular crowding could be achieved on artificial membranes. At and below protein concentrations corresponding to those found in the trypanosome surface coat, the diffusion coefficient of VSG is around 1.10±0.20 μm^2^ s^−1^ (SD, *N*⩾21 for all data points). Upon further increase in VSG concentration, the diffusion coefficient decreased significantly to 0.10±0.09 μm^2^ s^−1^ (SD, *N*=24) and the mobile fraction dropped below 40%. Thus, there exists a critical value that limits VSG mobility that is a prerequisite for VSG functionality. We have termed this the MCT. The MCT of VSG was reached at 1.5-fold of the VSG concentration on living trypanosomes (Fig. 1a,b). This result was confirmed with a second complete set of experiments (Supplementary Fig. 2A,B).

In accordance with the model of Evans and Sackmann, we used the measured drop in the VSG diffusion constant to calculate the viscosity of the dense VSG protein layer *μ*_VSG_ (see ‘Methods' section). We found *μ*_VSG_≈75 Pas, which is more than two orders of magnitude higher than the bulk viscosity of the artificial membrane. This result underpins the need for a new model describing diffusion of GPI-anchored proteins in dense environments such as the VSG coat or the extracellular matrix, where the popular SD model in the limit of *μ*_w_<<*μ*_m_ cannot be applied anymore.

Our findings clearly indicate that the trypanosome VSG coat operates rather close to its MCT. Nevertheless, this threshold is above the concentration of VSG on the trypanosome cell surface. Does this mean that the plasma membrane of trypanosomes could actually harbour more VSG molecules? It would indeed appear sensible for the parasites to be able to transiently adjust VSG density, for example in the course of antigenic variation, when two VSGs populate the cell surface at the same time. In fact, ectopic VSG overexpression transiently raises the overall amount of VSG to roughly 140% of wild-type levels. This resembles the MCT measured *in vitro* and FRAP measurements showed that the diffusion coefficient of VSGs on living parasites is significantly reduced (*P*=0.005, two-tailed and unpaired *t*-test) in the presence of excess VSG (Table 2). Thus, molecular crowding greatly influences VSG diffusion in artificial membranes and on living cells.

### Non-VSG proteins alter the crowding behaviour of the VSG

**Figure 2:**
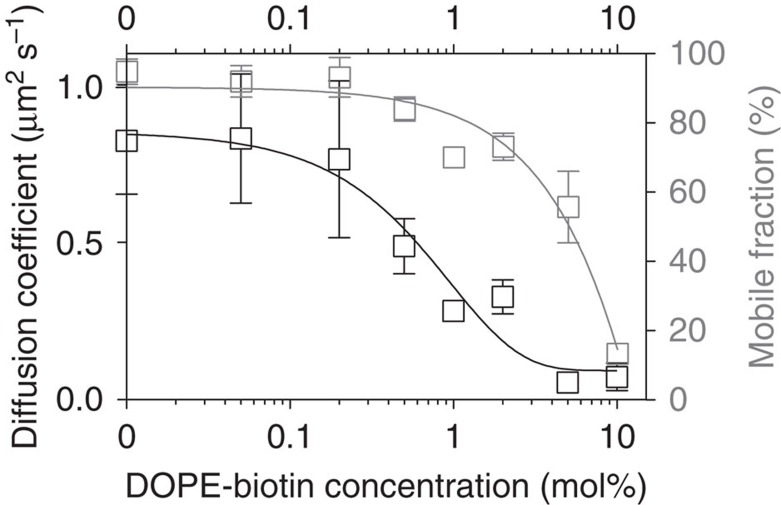
Influence of varying concentrations of membrane-bound NeutrAvidin on the diffusion of VSG in supported membranes. The black squares indicate VSG diffusion coefficients and grey squares represent the corresponding mobile fractions. Trend lines are inserted as a guide for the reader's eye. At high concentrations of non-VSG proteins (5–10% DOPE-biotin) the diffusion coefficients decrease more than 15-fold and VSG mobility is reduced to 20%. At low concentrations of non-VSG (0.1–1% DOPE-biotin) the diffusion coefficients are decreased fourfold, whereas the mobile fraction is reduced to 70%. Data are presented as means±s.d.

In contrast to the artificial VSG coats described above, the cell surface of living parasites features at least 5% of non-VSG proteins. Little is known about the nature of these additional proteins or their exact distribution on the trypanosome cell surface and, hence, it is impossible to measure or even adjust the amount of invariant proteins in *in vivo* experiments. We therefore developed a straightforward strategy to study the potential influence of heterologous proteins on VSG diffusion using the supported membrane system. Lipid bilayers with varying concentrations of biotinylated phospholipids (DOPE-biotin) were generated. The diffusion of VSG in doped and non-manipulated supported membranes was virtually identical, which excludes a changed diffusion behaviour due to the membrane lipid composition. The presence of non-VSG proteins was simulated by addition of NeutrAvidin, a small protein (MW=60 kDa) that strongly binds biotin. Strikingly, even comparatively small amounts of NeutrAvidin had a marked effect on VSG diffusion. Concentrations between 0.05 and 1 mol% DOPE-biotin reduced the diffusion coefficient of VSG to about one quarter of that observed in the absence of DOPE-biotin without having a comparable effect on the mobile fraction, which was only reduced from 90% to 75% (Fig. 2). When DOPE-biotin was added in saturating amounts, the calculated frictional coefficients *b*_s_ (see equations (3) and (4)) on VSGs before and after the addition of NeutrAvidin are *b*_s,before_= 4 × 10^7^ N s m^−3^ and *b*_s,after_= 8 × 10^9^ N s m^−3^, respectively. Thus, the occupation of free area with NeutrAvidin results in a significant increase in the frictional stress experienced by the VSG proteins.

**Figure 3:**
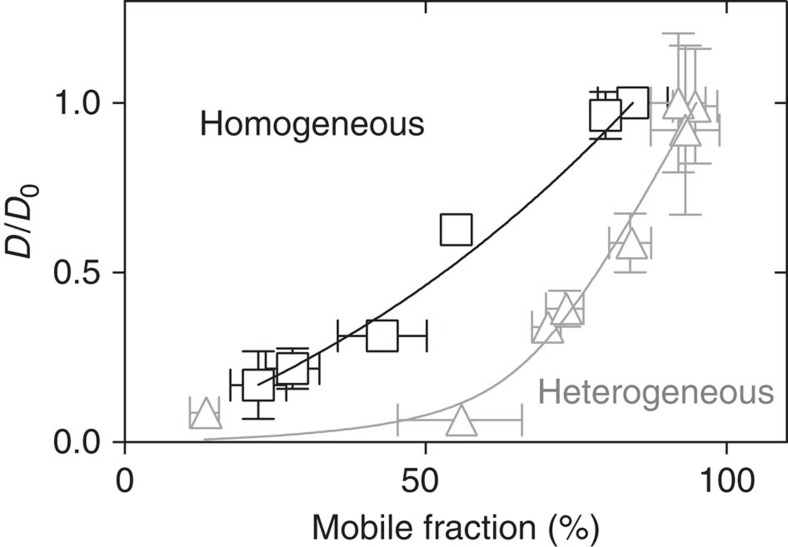
VSG diffusion depends on the density and homogeneity of the protein layer. Comparison of the diffusion coefficients and the corresponding mobile fractions in homogenous VSG (black squares) and VSG/NeutrAvidin layers (grey triangles, cf. Fig. 2). Diffusion coefficients (*D*) are normalized to the values for the most dilute situation (*D*_0_). The solid lines represent the fit according to equation (6). Data are presented as means±s.d.

By contrast, in a homogenous VSG-layer, that is, in the absence of NeutrAvidin, an equivalent reduction of the diffusion coefficient was accompanied by a decrease of the mobile fraction to 30% (Fig. 3). This is an interesting result, as it indicates that non-VSG proteins can exert a smaller effect on VSG mobility than the VSG itself. Thus, the MCT not only depends on the concentration but also on the physicochemical nature of the crowding agents. How does this translate to the *in vivo* situation? The range of concentrations of NeutrAvidin used in our experiments is comparable to the 5–10% of non-VSG proteins on the trypanosome cell surface. The NeutrAvidin-bound DOPE is smaller than VSG and its diffusion coefficient is three-fourth of that of the VSG. This could also hold true for invariant proteins on the trypanosome surface. These proteins are shielded by the surface coat and hence, should generally be smaller than a VSG. Furthermore, many invariant proteins are transmembrane proteins, which diffuse more slowly than GPI-anchored proteins^41^. Thus, the VSG/NeutrAvidin system emulates the parasite's surface coat with a good experimental accessibility, albeit with strongly reduced complexity. The fit of the data in Fig. 3 with equation (6) implies distinctly different *γ* values; in the presence of NeutrAvidin *γ*_inhom_=14±8 and VSG alone *γ*_hom_=0.5±0.5. This finding suggests that the NeutrAvidin molecules do indeed hinder the diffusion of VSGs and may explain why the diffusion coefficient of the VSG on living trypanosomes was found to be smaller than on homogenous, supported membranes: on living cells the non-VSG proteins slow down VSG diffusion, as they act as obstacles in the crowded environment of the VSG coat. Importantly, while those obstacles impair the diffusion coefficient, the overall fraction of VSGs that is mobile remains largely unaffected. This physical property of the VSG coat could prove to be critical for trypanosome survival in the mammalian bloodstream. The parasites remove VSG-bound host antibodies with the help of hydrodynamic drag forces that are generated on the cell surface by directional cellular movement. The hydrodynamic flow pushes antibody-bound VSGs against the swimming direction towards the flagellar pocket, where they are endocytosed and cleared from the circulation^42^. Since this hydrodynamic mechanism exerts an external force specifically on VSG-antibody complexes, the diffusion coefficient of the VSG is irrelevant, whereas the mobile fraction on the other hand becomes highly important. If VSG mobility were stalled, antibody clearance would lose its efficiency and the parasite its virulence.

### N-linked glycans facilitate membrane protein mobility

**Figure 4:**
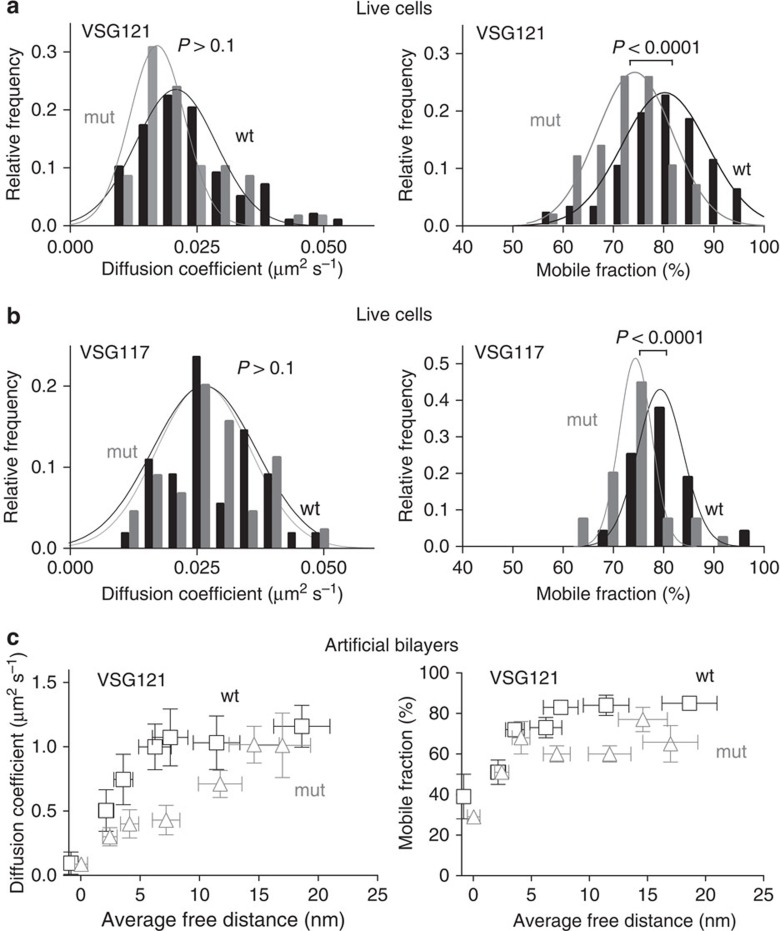
Impact of N-glycosylation on the diffusion of VSG. The relative frequency distributions for wild-type (wt) and N-glycosylation deficient (mut) VSG121 (**a**) and VSG117 (**b**). Frequency distributions were fitted with a Gaussian function. The diffusion coefficients do not differ significantly, whereas there is a significant change in the mobile fractions of the wt and mut VSG. (**c**) Impact of the lateral protein density of wt and N-glycosylation deficient (mut) VSG121 on their diffusion in artificial membranes. The error bars in *y* (diffusion coefficient) represent the standard deviation of the mean, while the error bars in *x* (average free distance) depict the range. *P* values determined by an unpaired *t*-test.

Nearly all VSGs that have been proven to be functionally intact are modified by post-translational addition of N-linked glycans. To test the possibility that N-glycosylation modulates VSG diffusion, we have removed the N-glycosylation sites of two VSGs by site directed mutagenesis. The transgenic cell lines produced an N-glycan free surface coat without any changes in cell morphology or growth (Jones *et al*., in preparation). While the diffusion coefficients of the VSG mutants were comparable to those of the respective wild-type proteins under *in vivo* conditions, their mobile fractions were significantly smaller (Fig. 4a,b). This means that N-glycosylation contributes to preserving high VSG mobility within the surface coat of living trypanosomes. Interestingly, both the diffusion coefficients of the mutant VSGs and their respective mobile fractions were reduced in the defined supported membranes system (Fig. 4c and Supplementary Fig. 2C,D). As a consequence of the lack of N-glycosylation, the MCT was shifted dramatically. VSG121 (wt) shows a MCT of between 5 and 7 nm average free protein distance, whereas the diffusion coefficient of its N-glycosylation mutant already begins to drop at larger distances (∼15 nm). The mobile fractions decrease to 30–40% with increasing protein density. Further, an additional set of experiments, wherein the concentration in the VSG layers was again quantified in means of cell surface equivalents, shows that the MCT of the N-glycosylation deficient mutant VSG is already reached at a onefold concentration (Supplementary Fig. 3).

**Figure 5:**
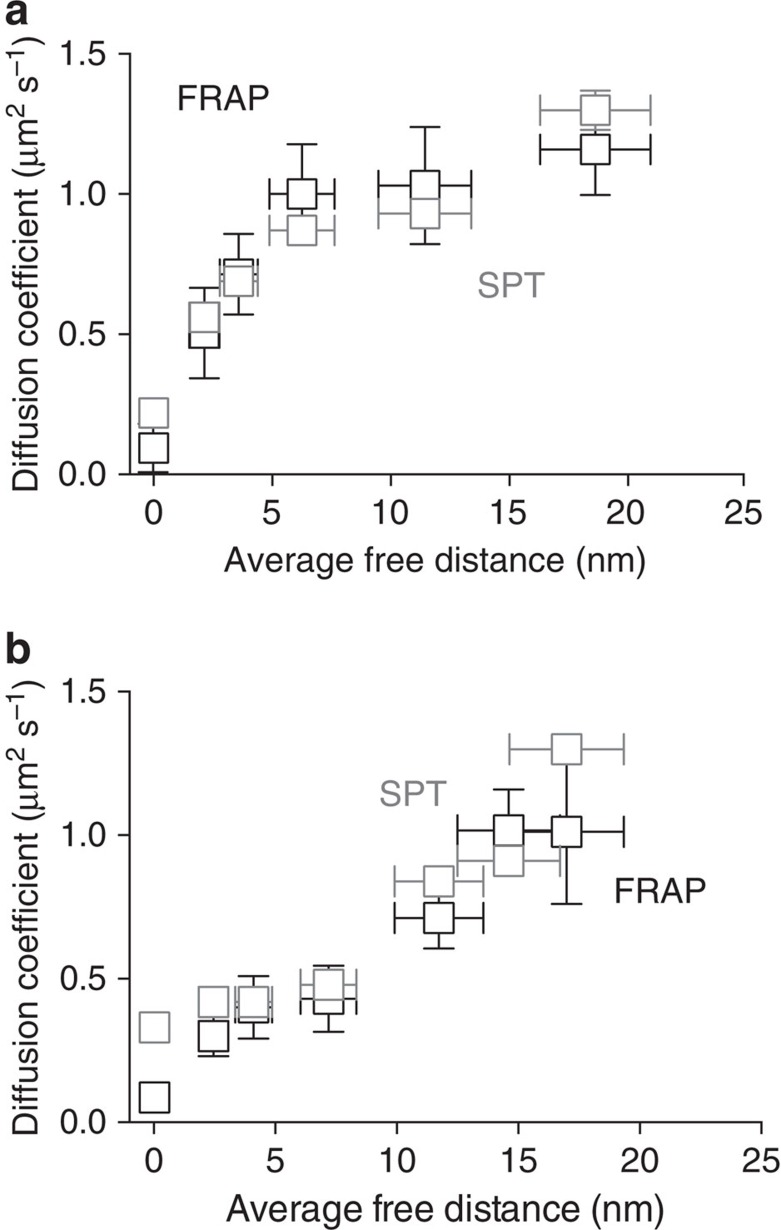
Comparison of VSG diffusion analysed by SPT or FRAP measurements. The impact of the lateral protein density in supported membranes on the diffusion of wild type (**a**) or N-glycosylation deficient VSG121 (**b**) was analysed using SPT (grey squares) and FRAP (black squares). The error bars in *y* (diffusion coefficient) represent the standard deviation of the mean, while the error bars in *x* (average free distance) depict the range. SPT, single-particle tracking.

To probe *in vitro* VSG diffusion on time- and length scales beyond the resolution of the FRAP technique (1 min and 1 μm, respectively), complementary single-molecule tracking experiments were implemented. To this end, the same SLBs as used in the FRAP measurements were further doped with trace amounts of VSG-ATTO 647N for single-molecule tracking on the millisecond timescale at a positional accuracy of 20±12 nm (SD, *N*>1.2 × 10^6^ localizations). For each condition a minimum number of 200 traces with a weighted mean trace length of 70 positions were analysed. Ensemble averaging allowed comparison with the FRAP results and yielded good agreement (see Fig. 5a,b). Close inspection of the individual traces revealed that the mobile fraction was >90% and independent of the VSG density throughout the phase space under investigation. However, as the coat densified an increasing fraction of the tracked VSGs exhibited confined diffusion in compartments well below the resolution limit of the FRAP technique (see Supplementary Fig. 4 for exemplary traces of free, confined and immobile VSGs). This fraction was generally larger for the mutant VSGs.

Therefore, we conclude, that N-glycosylation facilitates the dense packaging of VSG in 2D, probably by functioning as a structural ‘insulator' that decreases, for example, attractive interactions as suggested by Høiberg-Nielsen *et al*.^43^ for the three-dimensional situation. The ‘buffering' property of N-glycans could be essential during antigenic variation and the microevolution of new mosaic VSGs.

In conclusion, the above experiments prove that the part of the protein protruding from the membrane significantly influences diffusion and lateral mobility of GPI-anchored proteins. We suggest that for each membrane protein, a specific MCT exists that limits diffusion and hence influences protein function. This goes beyond the theoretical considerations of Saffman and Delbrück. The MCT describes that point at which the protein density increases the viscosity of the proteinaceous layer on the membrane beyond a critical value where diffusion is affected. This viscosity is certainly higher than that of water and thus contributes substantially to the lateral diffusion of membrane-anchored proteins. The cell surface coat of African trypanosomes operates surprisingly close to its MCT, with N-glycosylation of the VSG playing a major role.

Because of its greatly reduced complexity, the trypanosome system appears ideal for comparative studies on membrane protein diffusion. Furthermore, the finding that N-glycosylation influences the MCT of VSG diffusion might pave the way for new therapies against the deadly sleeping sickness. Any interference with the MCT of the VSG coat would unequivocally kill the parasites. This time the drug target would not be a particular protein or process, but the entire cell surface; it is hard to envisage how resistance against such a prospective drug could arise.

## Methods

Additional information is provided in Supplementary Methods section.

### Trypanosomes

*Trypanosoma brucei* bloodstream form (BSF) strains expressing VSG117, VSG121, VSG060, VSG221 or VSG118 were employed. N-glycosylation deletion mutants were generated based on the transgenic cell line 13-90 (ref. 44) by insertion of a mutated VSG121 or VSG117 coding sequence into the active VSG221 expression site via the construct pKD4 (ref. 45). Subsequently, the endogenous VSG221 gene was knocked out.

### Standard cultivation

*T. brucei* BSF were cultivated in HMI-9 medium, supplemented with 10% fetal calf serum, at 37 °C and 5% CO_2_. Unless otherwise stated, the maximum cell density was limited to 5 × 10^5^ cells per ml.

### Cell surface staining

A total of 10^7^ trypanosomes were washed three times in ice-cold TDB (5 mM KCl, 80 mM NaCl, 1 mM MgSO_4_, 20 mM Na_2_HPO_4_, 2 mM NaH_2_PO_4_, 20 mM glucose, pH 7.6), trypanosome density was adjusted to 1 × 10^8^ cells per ml, and cells were incubated with 10 μM ATTO 488 NHS-ester (ATTO-TEC GmbH, Siegen) for 15 min on ice in the dark. To remove unbound dye, the cells were washed three times with ice-cold TDB^46^.

### Immobilization of trypanosomes

A total of 10^7^ fluorescently labelled trypanosomes were suspended in 20 μl of TDB. For immobilization 3 μl of the cell suspension were added to 5 μl of 10% (w/v) type A gelatin (from porcine skin; Sigma-Aldrich, Steinheim) in PBS, pH 7.8, warmed to 37 °C. The trypanosome-gelatin mixture was pipetted between two cover slips and mounted in a temperature-controlled sample holder, which was then cooled to 20 °C.

### High-density cultivation of trypanosomes

Cells were grown to a cell density of 8 × 10^5^ cells per ml; then one volume of fresh HMI-9 medium, supplemented with 30% fetal calf serum, was added and incubation was continued in conical glass flasks with constant orbital shaking (80 r.p.m.). This cultivation procedure supports exponential growth of BSF to a maximum cell density of 1 × 10^7^ cells per ml.

### Purification and fluorescence labelling of membrane form VSG

The purification of membrane form VSG (mfVSG) was carried out as published by Clarke *et al*.^39^. Cell pellets were washed three times in ice-cold TDB, proteins were extracted using a 0.1% trifluoroacetic acid solution on ice and subsequently purified via high-performance liquid chromatography. Purified mfVSG was labelled with ATTO 488 NHS-ester (DOL=1–2) or ATTO 647N NHS-ester (DOL<1, ATTO-TEC GmbH, Siegen), suspended in vesicle buffer (20 mM Tris-HCl, pH 7.4, 50 mM NaCl and 0.5 mM CaCl_2_) and stored at −20 °C. For more details please see Supplementary Methods.

### Preparation of hydrophilic glass cover slips

Glass cover slips (Hecht Assistent, No. 1, 24 mm diameter) were cleaned by sequential sonication in acetone, ethanol and methanol for 15 min each. After washing in deionized water the cover slips were soaked in a mixture of 1 : 1 : 5 (v/v/v) 30% ammonia : 30% hydrogen peroxide : deionized-H_2_O for 30 min at 60 °C, followed by rigorous rinsing with deionized-H_2_O and drying at 70 °C. Hydrophilic glass cover slips were stored in a vacuum desiccator to avoid contamination with dust and were used within 2 days.

### Incorporation of mfVSG into lipid bilayers

Lipid bilayers were prepared from DOPE-biotin (1,2-dioleoyl-sn-glycero-3-phosphoethanolamine-N-cap-biotinyl) and/or SOPC (1-stearoyl-2-oleoyl-sn-glycero-3-phosphocholine, Avanti Polar Lipids Inc., Alabaster) by fusion of small unilamellar lipid vesicles on hydrophilic glass substrates (for details see Supplementary Methods section). Following preparation of the lipid bilayers, 0.2 nmol of fluorescently labelled mfVSG was applied to the sample chamber and incubated under light protection at 37 °C for 1 h. Finally, the sample was washed with 50 ml of vesicle buffer. For the preparation of heterogeneous protein layers, consisting of mfVSG and differing concentrations of NeutrAvidin (Invitrogen, Darmstadt), varying amounts of DOPE-biotin were added to the small unilamellar lipid vesicles (for details see Supplementary Methods section).

### mfVSG reconstitution and determination of lipid-to-protein ratio

Labelled mfVSGs were reconstituted into liposomes by detergent extraction and subsequent removal of non-incorporated mfVSG by ultracentrifugation and dialysis^47^ (for detailed information see Supplementary Methods section). Three aliquots of proteoliposomes were used in a modified total phosphorus assay (Anderson and Davis^48^; Bartlett^49^) to quantify the lipid concentration. The mfVSG concentration was determined photometrically and the lipid-to-protein ratio was calculated. The molar extinction coefficient of VSG at 280 nm was calculated by ProtParam based on the amino acid sequence of the mature VSG^38^.

SOPC monolayers were deposited on hydrophilic glass cover slips by Langmuir-Blodgett transfer using a Langmuir film balance (Kibron Inc., Finland). SOPC was dissolved in chloroform and spread on the water sub-phase of the Langmuir trough. The lipid monolayers were transferred to the glass cover slips at a constant surface pressure of 32 mN m^−1^.

The spacing of mfVSG on the artificial membranes was adjusted by diluting proteoliposomes of known lipid-to-protein ratio with defined amounts of protein-free liposomes. Pure or diluted proteoliposome samples were added to SOPC monolayers, incubated for 2 h at 37 °C and washed with 50 ml of vesicle buffer. For single-molecule microscopy experiments 0.1 pmol ATTO 647N-labelled mfVSG were then applied to preformed protein layers and incubated for 5–15 min at 37 °C. Finally, the samples were washed again with 50 ml of vesicle buffer.

### Absolute quantification of mfVSG in bilayers

To determine the average free distance, *d*, between the VSG dimers the dimensions of the N-terminal domain of the known structures (VSG221 (PDB-ID: 1VSG) and ILTat1.24 (PDB-ID: 2VSG)) were measured at its greatest width (depth, *d*_VSG_) and smallest width (width, *w*_VSG_). With 6.5 nm, the largest value for *d*_VSG_ was found in VSG221 whereas ILTat1.24 displayed the smallest width with *w*_VSG_=4.5 nm.

Using these dimensions, the average free distance, *d*, between VSG dimers within the SLB was calculated from the measured lipid-to-protein ratio, *lpr*, as follows:


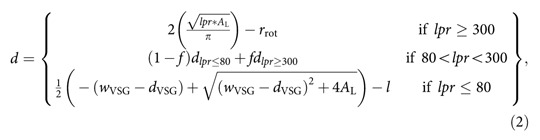


where *A*_L_=0.6 nm^2^ denotes the area per SOPC head group. At low protein densities (*lpr*⩾300) the VSG dimers were assumed to be able to rotate freely with *r*_rot_=*(d*_*VSG*_^*2*^*+w*_VSG_^*2*^)^*1/2*^ on a honeycomb lattice. The area of the rotating dimer is equivalent to ∼80 × *A*_L_. In the limit of high protein densities (*lpr*≤80) this rotation is assumed to be sterically impeded and the VSG dimers align on a rectangular grid. This transition takes place at intermediate protein densities (80<*lpr<*300). In this regime, *d* is calculated from the weighted mean of the two limiting cases. The weighting factor *f*=(*lpr*–80)/219. The upper *lpr* threshold was chosen to ensure a smooth transition in the *lpr*-to-*d* conversion.

### Relative quantification of mfVSG in proteoliposomes

The density of mfVSG in artificial membranes was expressed as trypanosome cell surface equivalents. For this purpose, pure or diluted proteoliposome samples were added to SOPC monolayers and three aliquots of the supernatant were collected immediately or after 2 h of incubation at 37 °C. These two samples, A and B, were used to quantify the amount of mfVSG incorporated into the artificial membrane as explained in detail in Supplementary Methods section.

### Fluorescence microscopy and FRAP

A fully automated inverted wide-field fluorescence microscope (TILL Photonics, Gräfelfing) was equipped with a Yanus digital scan head, 473 and 561 nm (Cobolt Inc., Solna) lasers, the appropriate emission and excitation filter cubes, Polychrome V (TILL Photonics) and a CCD camera (Sensicam, pixel size 6.4 μm, PCO, Kelheim). FRAP experiments were performed with a Nikon × 60 objective lens (NA 1.45). The laser was used for irreversible photobleaching of the region of interest. Polychrome V illumination was used to record pre and post-bleach frames. All equipment was controlled with the ‘Live Acquisition' software (FEI) and images were analysed with the ‘Offline Analysis' software packages (FEI). Protein diffusion was quantified by FRAP measurements. For measurements on living trypanosomes, line-FRAP was performed and data were analysed according to Phair *et al*.^50^. On artificial membranes circular regions of interest were bleached and diffusion was analysed according to Soumpasis *et al*.^51^.

### Analysis of lateral diffusion

We calculated the frictional drag in artificial, supported membranes from the measured VSG diffusion coefficients in accordance with the approach of Evans and Sackmann which relates the diffusion coefficient *D* with the dimensionless radius *ɛ* (refs 10, 15, 52, 53):


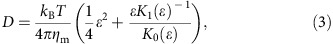


where *k*_B_ is the Boltzmann constant, *T* the temperature and *K*_1_ and *K*_0_ modified Bessel functions of second kind with first or zeroth order, respectively. *η*_m_ is the membrane surface viscosity (bulk viscosity times membrane thickness). *ɛ* is given by


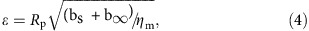


where *R*_p_ is the radius of the diffusing particle, *b*_s_ the friction coefficient with the substrate and *b*_∞_ the friction coefficient with the ambient medium, either water or the VSG protein layer. In the dilute regime of low VSG concentrations the friction with the substrate is dominating over the friction with the ambient medium (*b*_s_>>*b*_∞_). The opposite is true in the regime of high VSG concentrations. Thus, *b*_∞_ can be determined from the diffusion coefficient measured at high VSG concentrations and the corresponding viscosity of the protein layer can be calculated according to


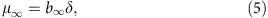


where *δ* is a length which characterizes the penetration of the membrane flow field into the third dimension. Here, we assume that the membrane flow field will vanish over the full height of the protein layer, thus *δ*=15 nm (refs 29, 54).

Since equation (3) cannot be inverted easily, we employed a graphical solution to extract *ɛ* and subsequently calculated *b*. During the experiments the temperature was kept constant at *T*=20 °C. The relevant size of the diffusing particle in the membrane was determined by the two GPI anchors of the VSG dimer (*R*_p_=1 nm). We use *η*_m_=0.65 × 10^−9^ Pas. To make an educated guess of the 2D viscosity of a SOPC membrane at 20 °C, we linearly interpolated the reported values of DOPC at 10 °C (0.85 × 10^−9^ Pas) and 45 °C (0.16 × 10^−9^ Pas) (ref. 52). Comparison of this approximation with the known relationship between viscosity and temperature for water^55^ and polymers^56^ results in an error of less than 50% which does not influence the reported order of magnitude of the frictional coefficient.

### The effect of obstacles

The diffusion in the presence of mobile obstacles was simulated by Saxton^14^, based on Tahir-Kheli^12^ and van Beijeren *et al*.^13^,


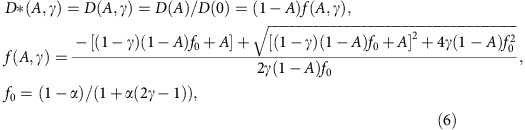


where *A* is the area fraction of the obstacles and *α* a lattice-specific constant (*α*=0.5, corresponding to a honeycomb lattice). *γ* is the ratio between the jump rate of a diffusing particle and the jump rate of obstacles. We chose this model to analyse the diffusion of VSG in the presence and absence of NeutrAvidin. In our experiments, we interpret the measured immobile VSG fraction as the area fraction of mobile obstacles. It should be noted, that the immobile fraction is only immobile on the timescales used for the experiment not, however, at longer timescales. Thus, the VSG is tracer and obstacle at the same time while NeutrAvidin is only a modulating factor without an explicit analogue in the model. The fit is presented in a plot of the mobile fraction *mf* versus *D/D*_*0*_ (see Fig. 3). To this end, the data of the immobile fraction *imf* are converted via *mf*=1–*imf*.

### Single-molecule tracking

Image analysis was done as described before^57^,^58^ using programs written in MatLab (Mathworks Inc., USA). In brief, each frame of a single-molecule movie was corrected for static background by low-spatial frequency filtering. The *x*/*y* positions of the individual ATTO 647N molecules were determined by fitting a 2D-Gaussian to the acquired intensity distributions. This data set was further filtered by comparison to the single-molecule footprint of ATTO 647N (intensity, width) and detection error thresholds. Trajectories were obtained from the single-molecule positions using a probabilistic algorithm that calculates the probability of a particle with diffusion coefficient *D*_in_ to reappear at a different position in the subsequent frame. A transitional matrix is built up, which includes the probabilities of all possible connections between all molecules in neighbouring frames. From these a combination of connections is chosen in such a way that the total probability is maximized. For more details on how to choose *D*_*in*_ properly, see the Supplementary Methods section and the Supplementary Fig. 5.

Only trajectories with 20 or more steps were analysed further. They were categorized as mobile or immobile by comparison with the achieved localization precision, *σ*. An immobile particle is characterized by *msd*_immob_=4*σ*^2^.

Starting from the trajectories of mobile particles, the mean squared displacement, *msd*, was calculated as a function of time, *t*. Both the *msd* and the time between frames were subsequently corrected for limitations in the time and spatial resolution^25^,^59^,^60^.


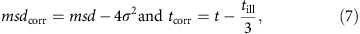


where *t*_ill_ denotes the illumination time. *D*_out_ was calculated as one-fourth of the slope of the *msd*_corr_(*t*_corr_) curve resulting from a linear fit to the five first data points.

### Data availability

All the relevant data that support the findings of this study are in principle available from the corresponding author upon request.

## Additional information

**How to cite this article:** Hartel, A. J. W. *et al*. N-glycosylation enables high lateral mobility of GPI-anchored proteins at a molecular crowding threshold. *Nat. Commun.* 7:12870 doi: 10.1038/ncomms12870 (2016).

## Supplementary Material

Supplementary Information Supplementary Figures 1-5, Supplementary Table 1, Supplementary Note 1, Supplementary Methods and Supplementary References

## Figures and Tables

**Table 1:** Diffusion of various VSGs on live cells and on supported membranes.

**Live cells**				**Supported membranes**			
**VSG (no. of N-Glycans)**	**Diffusion coefficient**±**s.d. (μm^2^ s^−1^)**	**Mobile fraction**±**s.d. (%)**	***N***	**VSG (no. of N-Glycans)**	**Diffusion coefficient**±**s.d. (μm^2^ S^−1^)**	**Mobile fraction**±**s.d. (%)**	***N***
121 WT (1)	0.024±0.010	79±9	110	121 WT (1)	1.0±0.3	94±6	61
121 MUT (0)	0.021±0.010	71±7	56	121 MUT(0)	0.7±0.2	95±8	56
117 WT (1)	0.028±0.009	80±6	43				
117 MUT (0)	0.030±0.013	75±6	36				
060 (2)	0.021±0.013	74±7	40	060 (2)	0.9±0.2	94±5	41
221 (2)	0.021±0.008	76±6	28	221 (2)	0.7±0.2	94±8	58
118 (3)	0.027±0.008	78±8	25	118 (3)	0.7±0.2	97±2	41

VSG, variant surface glycoprotein.

**Table 2:** Effect of VSG overexpression on the diffusion of VSG on live cells.

**Relative degree of VSG overexpression**	**Diffusion coefficient±s.d. (μm**^**2**^** s**^**−1**^**)**	**Mobile fraction**±**s.d. (%)**	***N***
1	0.020±0.005	78±11	18
1.2	0.019±0.008*	79±11	28
1.4	0.016±0.005^#^	77±11	29

VSG, variant surface glycoprotein.

*P* value of the diffusion coefficients of a two-tailed and unpaired *t*-test compared with a degree of overexpression of 1: P*>0.03, *P*^#^=0.005
