# Supplementary material for: N-glycosylation enables high lateral mobility of GPI-anchored proteins at a molecular crowding threshold
Source: Nat Commun. 2016 Sep 19;7:12870. doi: 10.1038/ncomms12870 (PMC5031801; doi:10.1038/ncomms12870)
Supplement: Supplementary Information — Supplementary Figures 1-5, Supplementary Table 1, Supplementary Note 1, Supplementary Methods and Supplementary References [file ncomms12870-s1.pdf]

Supplementary Information:

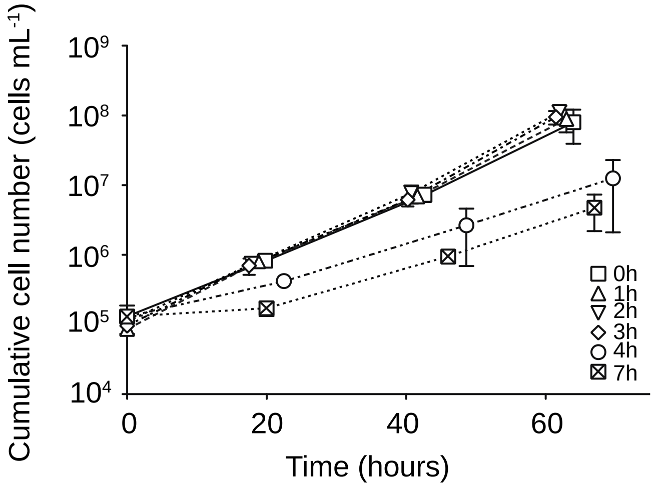

Supplementary Figure 1. Re-cultivation of bloodstream form trypanosome cells after gelatin immobilization. Cells were immobilized at 20 °C for the times indicated and subsequent cell growth was monitored for 72 hours in HMI-9 medium at 37 °C.

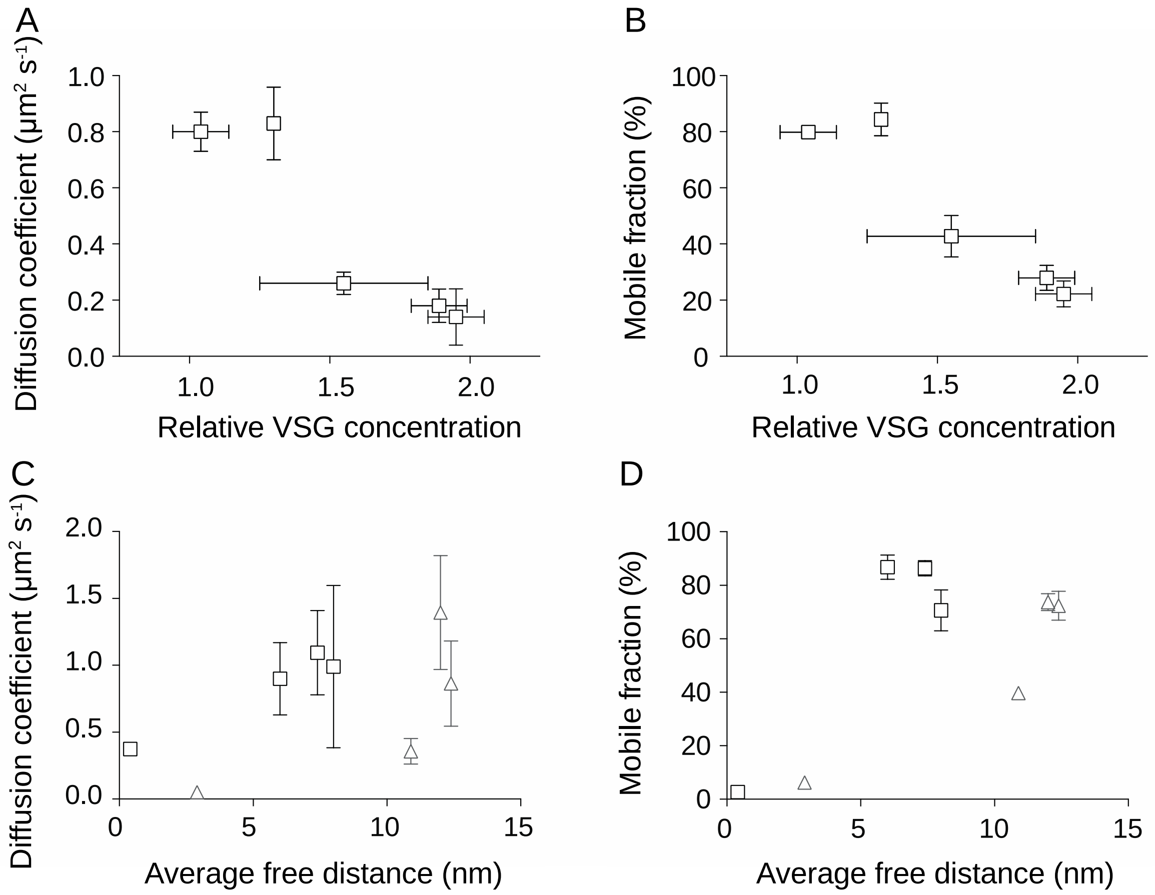

Supplementary Figure 2. Confirmation of results. Determination of the molecular crowding threshold (MCT) and the effect of N-glycosylation on VSG diffusion by a separate set of experiments. VSG diffusion coefficient (A) and mobile fractions (B) decrease dramatically between a 1.2 - 1.5-fold VSG-concentration. The error bars in y (diffusion coefficient or mobile fraction) represent the standard deviation of the mean while the error bars in x (relative VSG concentration) depict the range. The viscosity of the VSG protein layer was calculated from the drop in the diffusion coefficient to be ≈ 45 Pas. Confirmation of the effect of VSG N-glycosylation on its diffusion. Diffusion coefficients (C) and mobile fractions (D) of wild type (black squares) and N-glycosylation deficient (grey triangles) VSG. Data are presented as means ± SD.

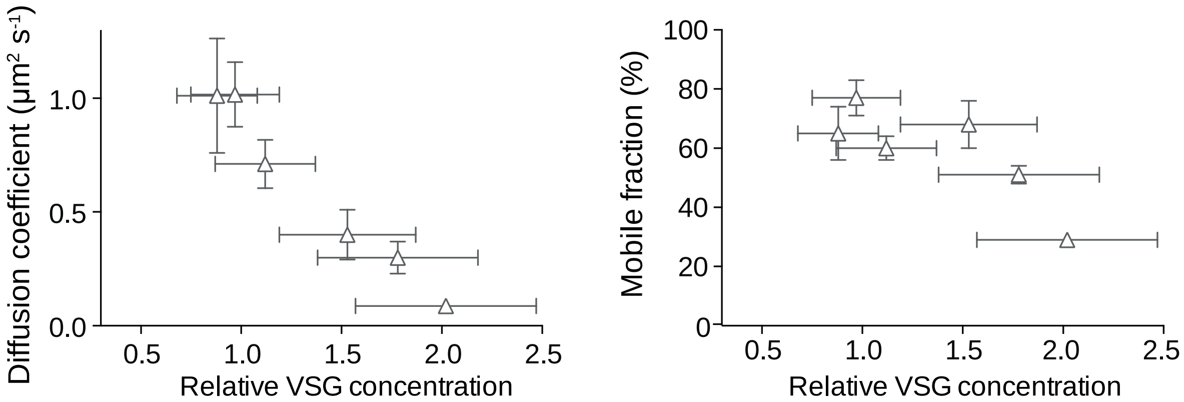

Supplementary Figure 3. **N-glycosylation deficient VSG coats exhibit a decreased molecular crowding threshold.** The impact of lateral protein density on the diffusion of VSG was quantified in supported membranes. Diffusion coefficients (left panel) and mobile fractions (right panel) are displayed as a function of the VSG concentration in relation to the concentration on the trypanosome cell surface. A relative concentration of 1 corresponds to the concentration *in vivo.* The error bars in y (diffusion coefficient) represent the standard deviation of the mean while the error bars in x (relative VSG concentration) depict the range.

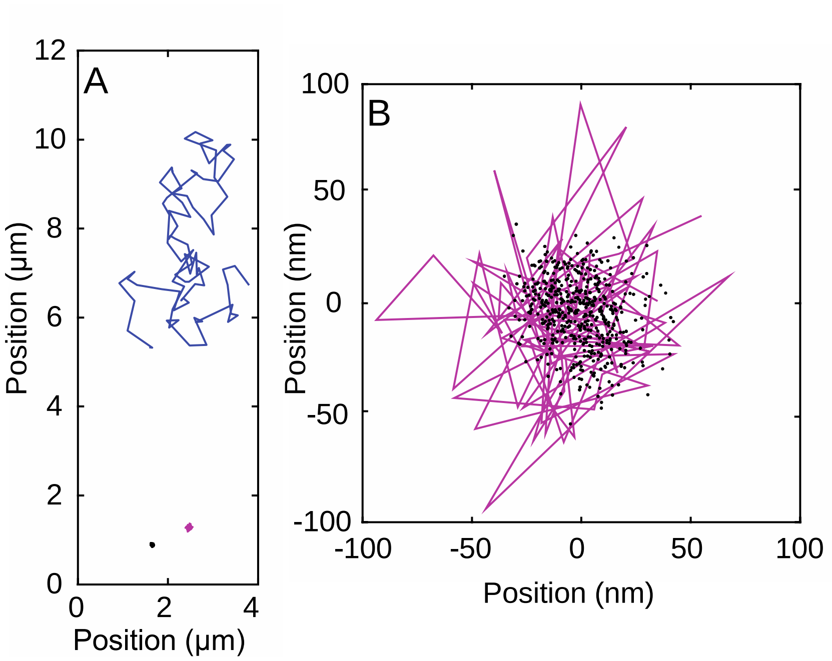

Supplementary Figure 4. Single-molecule traces of VSGs diffusing in model membranes. Exemplary traces of free (blue), confined (purple) and immobile (black) VSGs (A). Comparison of the confined and the immobile trace. The mid-points of the traces were centered for better comparability (B).

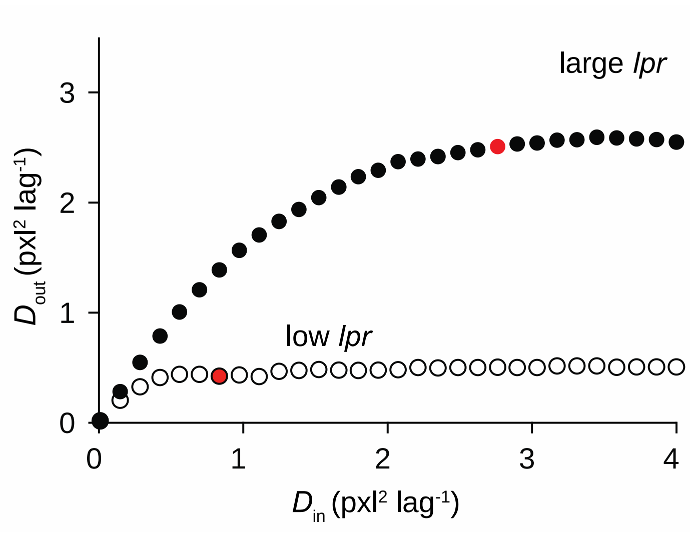

Supplementary Figure 5. Exemplary D_{in} versus D_{out} analysis for N-glycosylation deficient VSG121 reconstituted proteoliposomes with large *lpr* (closed circles) and low *lpr* (open circles). Red circles indicate respective D_{in} values used for further data analysis.

Supplementary Table 1. Diffusion coefficients and mobile fractions of VSG on supported membranes before and after addition of 6 % gelatin

	Diffusion coefficient ± SD ($\mu m^2 s^{-1}$)	Mobile fraction ± SD (%)	N
PBS	0.9 ± 0.2	96 ± 1	12
PBS + 6 % gelatin	1.2 ± 0.1	99 ± 1	12

Supplementary Note 1

Gelatin is a suitable substrate for immobilizing trypanosomes under physiological conditions

We found that a final concentration of approx. 6 % (w/v) type A gelatin from porcine skin in TDB or PBS is well suited for immobilization of trypanosome BSF at 20 °C. Trypanosomes were immobilized for different periods of time and subsequently re-cultivated to test the effect of the gelatin solution on cell viability. For this purpose, triplicates of 5×10^6 trypanosomes in 100 µL TDB were mixed with four volumes of 10 % gelatin solution and incubated at room temperature for either 0, 1, 2, 3, 4 or 7 hours. After incubation the cell-gelatin solution was heated to 37 °C. 100 µL of the cell-gelatin solution (1×10^6 cells) was suspended into a total volume of 10 mL HMI-9 medium. Subsequent cell growth was monitored for 72 hours using the standard cultivation procedure (Supplementary Fig. 1).

Diffusion of the VSG is not impaired by gelatin

We analyzed the effect of 6 % (w/v) gelatin on the diffusion of VSG on supported membranes. For this purpose, VSGs were incorporated into supported membranes that had been spread on glass cover slips. Diffusion of VSGs was analyzed by FRAP in the presence and absence of 6 % (w/v) gelatin in PBS at 20 °C. Neither the diffusion coefficient nor the mobile fraction of VSG were reduced in the presence of solid gelatin (Supplementary Table 1).

Supplementary Methods

Constructs and transgenic trypanosomes

For the generation of VSG121 and VSG117 N-glycosylation deficient cell lines VSG N-glycosylation signal peptides (N-X-S/T; X not P) were destroyed by site directed mutagenesis. VSG121 was mutated in position 434 of the mature protein to replace the endogenous threonine by alanine. VSG117 was mutated in position 422 of the mature protein to substitute threonine by alanine.

VSG overexpression

Overexpression of VSG was carried out using the tetracycline inducible cell line $221^{ES}.121^{tet}$ [1]. In this cell line a VSG121 coding sequence was inserted into the ribosomal spacer region of the transgenic cell line 13-90 via the plasmid pLEW82v4. The ectopic VSG121 was under control of a tetracycline-inducible T7-Promotor. The cell line $221^{ES}.121^{tet}$ was cultivated in the presence of 2.5 µg mL^{-1} G418, 5 µg mL^{-1} hygromycin and 1 µg mL^{-1} phleomycin.

Purification and fluorescence labeling of VSG

Typically, 5×10^9 trypanosomes were washed three-times with 50 mL of ice-cold TDB, followed by resuspension in 0.5 mL PBS and addition of 0.5 mL 0.2 % (v/v) trifluoroacetic acid (TFA) on ice. After centrifugation at 5000 × *g* and 4 °C for 5 min the VSG containing supernatant was collected. The extraction was repeated two times with 1 mL 0.1 % (v/v) TFA solution to obtain maximum yield. Insoluble material was removed by centrifugation at 15,000 × *g* and 4 °C for 15 min.

The protein extract was loaded onto a Perfectsil C3 rp-HPLC column (MZ-Analysetechnik, Mainz) in a Dionex (Thermo Scientific, Sunnyvale) HPLC system, equilibrated with solvent A and eluted with a stepwise gradient of solvents A and B (A: 0.1 % TFA in water; B: 0.1 % TFA, 90 % 2-propanol, 10 % water; gradient: 0-5 min 100 % A, 0 % B; 5-15 min 65 % A, 35 % B; 15-25 min 65 % A, 35 % B; 25-65 min 40 % A, 60 % B; flow rate: 4 mL/min; temperature: 37 °C). Fractions containing mfVSG were pooled, lyophilized and stored at -20 °C.

Purified mfVSG was resuspended in 0.1 M sodium bicarbonate buffer, pH 8.4 to a final protein concentration of approximately 2 mg mL^{-1}. Labeling with ATTO 488 NHS-ester (ATTO-TEC GmbH, Siegen) was done using the manufacturer’s protocol for 1 hour at room temperature under constant shaking and light protection. Free

label and labeled proteins were separated from each other using a PD-10 column (GE Healthcare, Solingen), equilibrated with 20 mL of vesicle buffer (20 mM Tris-HCl, pH 7.4, 50 mM NaCl and 0.5 mM $CaCl_2$).

Incorporation of mfVSG into lipid bilayers

SOPC and DOPE-biotin were dissolved in chloroform, dried by a stream of nitrogen-gas and rehydrated in vesicle buffer (20 mM Tris-HCl, pH 7.4, 50 mM NaCl and 0.5 mM $CaCl_2$) under vigorous shaking to yield a final lipid concentration of 1 mM. Vesicle formation was triggered by sonication until a translucent lipid suspension was formed. A hydrophilic glass cover slip was mounted in the sample chamber and as a rule 1 mL SUV solution was applied. After incubation for 1 hour at 37 °C, the sample was washed with 50 mL of vesicle buffer.

For the preparation of heterogeneous protein layers, consisting of mfVSG and varying concentrations of neutravidin (Invitrogen, Darmstadt), increasing amounts of DOPE-biotin were added to the SUVs. After spreading of the doped SUVs onto hydrophilic glass cover slips, fluorescently labeled mfVSG was added and the diffusion was measured. Then 5 nmol of neutravidin in vesicle buffer were added to the sample chamber followed by an incubation period of 1 hour at 37 °C, after which unbound neutravidin was removed by washing in vesicle buffer.

Reconstitution of mfVSGs into proteoliposomes

SOPC and mfVSG were mixed with 1 % (w/v) sodium deoxycholate in vesicle buffer at a molar lipid-to-protein ratio of 40 to achieve the highest possible packing of mfVSG in proteoliposomes. The mixture was agitated in the dark for 30 min at 30 °C, followed by 90 min incubation at 37 °C. Sodium deoxycholate was removed by incubating two times each with 100 mg SM-2 Biobeads for 60 min at 37 °C. Proteoliposomes and non-incorporated mfVSG were separated by ultracentrifugation in a four-step sucrose gradient (2 M, 1.2 M, 0.8 M, 0.4 M in vesicle buffer) at 120.000 × *g* for 18 h at 4 °C. The mfVSG containing proteoliposomes were collected from the sucrose gradient and dialyzed two or three times for at least 8 h against vesicle buffer.

Relative quantification of mfVSG in bilayers

Trypanosomes from an exponentially growing culture were washed three times with

TDB. Triplicates of whole cell protein extracts were dotted in a six step linear dilution series onto a nitrocellulose membrane ($1.3 \times 10^3 - 8.4 \times 10^4$ cells). The pure or diluted mfVSG proteoliposome samples A (before incubation on the SOPC monolayers) and B (after 2 h of incubation) were spotted onto the same nitrocellulose membrane in triplicates. VSG was detected on the nitrocellulose membrane with specific primary antibodies and according secondary antibodies using a LI-COR ODYSSEY IR Scanner (LI-COR, Lincoln). The linear dilution series and mfVSG samples A and B were used to calculate the relative amount of mfVSG in the artificial membrane expressed in trypanosome cell surface equivalents. The mean surface area of a bloodstream form trypanosome (BSF) (144 μm^2) [2] and the area of the supported membranes (4×10^8 μm^2) were used to compare the VSG density on supported membranes with the density on the trypanosome surface.

FRAP of mfVSG on cells

Line-FRAP measurements of labeled trypanosomes were performed at a constant temperature of 20 °C. Ten pre-bleach and 100 post-bleach images were recorded at 2 fps. VSG diffusion half-life (τ) and mobile fractions were determined using a single exponential function and data normalization according to Phair *et al.* [4]. The width (2x) of the lines bleached on the cell surface was determined by plotting the intensity profiles directly after bleaching. Diffusion coefficients were calculated by $D = (x)^2 / (4\tau)$. The diffusion coefficients and mobile fractions are means ± standard deviation. The data were statistically validated with an unpaired and two tailed t-test (GraphPad Prism 5.0f).

FRAP of mfVSG on supported membranes

The diffusion coefficient and mobile fraction of the mfVSG on artificial membranes was determined by FRAP at a temperature of 20 °C. Typically 10 pre-bleach and 100 post-bleach frames were recorded at 2 sec per frame and 5 µm bleach spot radius. Data analysis was performed according to Soumpasis *et al.* [5].

Single-molecule Tracking

As the algorithm requires a starting guess for D_{in}, which may also influence the resulting D_{out}, a D_{in} versus D_{out} analysis was performed for 30 consecutive D_{in} values

in the range of 0.01 - 4 (px^2 lag^{-1}). D_{in} was then chosen in a range where D_{out} was independent of D_{in} (Supplementary Fig. 5).

Supplementary References

1. Batram, C. *et al.* Expression site attenuation mechanistically links antigenic variation and development in Trypanosoma brucei. *eLife Sciences* **3,** e02324 (2014).
2. Grünfelder, C. G. *et al.* Accumulation of a GPI-anchored protein at the cell surface requires sorting at multiple intracellular levels. *Traffic* **3,** 547–559 (2002).
3. Harrington, J. M. J. *et al.* Novel african trypanocidal agents: membrane rigidifying peptides. *PLoS One* **7,** e44384 (2012).
4. Phair, R. D., Gorski, S. A. & Misteli, T. Measurement of dynamic protein binding to chromatin in vivo, using photobleaching microscopy. *Methods Enzymol.* **375,** 393–414 (2004).
5. Soumpasis, D. M. Theoretical analysis of fluorescence photobleaching recovery experiments. *Biophys. J.* **41,** 95–97 (1983).
